# Enhancing healthcare equity by using open-source pediatric medical devices in low resource settings: An exploratory international survey of pediatric clinicians

**DOI:** 10.1371/journal.pone.0334108

**Published:** 2025-10-24

**Authors:** Andrew G. Wu, Ryan C.L. Brewster, Ryan W. Carroll

**Affiliations:** 1 Division of Critical Care Medicine, Boston Children’s Hospital; Harvard Medical School, Boston, Massachusetts, United States of America; 2 Department of Pediatrics, Boston Children’s Hospital; Harvard Medical School, Boston, Massachusetts, United States of America; 3 Division of Pediatric Critical Care Medicine, MassGeneral Brigham Hospital for Children, Harvard Medical School, Boston, Massachusetts, United States of America; Touro University California College of Pharmacy, UNITED STATES OF AMERICA

## Abstract

Children in low-resource settings suffer from a high burden of treatable diseases that could be addressed with contextually appropriate technologies. However, numerous barriers to providing such technology to children in these settings exist. We propose that using open-source medical devices, where any qualified operator can freely make, modify, or distribute a product, may be a viable strategy to increase access to medical therapies in low-resource settings. However, given the novelty of open-source models, we sought to conduct an exploratory global survey on the perspectives and opinions of medical providers on the feasibility of this approach. Among 101 surveys completed by providers representing 34 countries, we found that the majority (89%) of respondents lacked experience working with open-source devices in low-resource settings; many respondents felt comfortable with providing an open-source pediatric medical device in a low-resource setting; lack of funding was the most significant barrier to successfully deploying these technologies; locally identified need was the most important factor to consider when conducting such projects; and respondents from the USA found no ethical issues with implementing open-source devices in low-resource settings, but respondents from outside the USA did find ethical issues with the same work. Our survey shows that most respondents in relevant specialties did not have experience working in either pediatric global health or with open-source medical devices in low-resource settings. Our survey may have revealed a potential unexplored frontier in addressing inequities in health care by enhancing access to equipment and technologies in areas of the world with the highest burdens of treatable pediatric disease, while also identifying ethical and cultural obstacles that warrant consideration.

## Introduction

Low-resource settings (LRS), which can exist in countries of any income level, are often thought of as communities where constraints on finances, health literacy, medical infrastructure and delivery, or social and human resources contribute to inequities of and limit the potential for optimal health outcomes [1]. Congruently, children living in LRS face a disproportionately high burden of treatable deaths, infections, and malnutrition along with a rising incidence of non-communicable diseases [2]. Clinically and contextually appropriate medical equipment and technologies are critical tools used by healthcare providers to address these challenges [3]. However, many products targeting the pediatric population fail to sustainably scale due to poorly defined pathways for development and commercialization, especially in LRS [4,5].

The barriers to designing and marketing medical technologies for children are multifactorial, and include a small market size, a wide range of rapid changes in anatomy and physiology, and the legal and ethical mandates surrounding testing products for children – and these challenges are magnified in LRS [5–7]. Lack of consistent electricity is a common problem faced and can render many types of equipment unreliable or even dangerous to use with children. Furthermore, implementing and maintaining service contracts for imported devices are nearly impossible to honor, leading to abandoned equipment when malfunctions occur [8]. In addition, it can be difficult for national regulatory systems to engage international stakeholders who are beholden to external standards.

Designing devices and tools specifically for LRS, focusing on maximizing accessibility and sustainability, may overcome potential barriers. One such approach is to utilize the “open-source” process, a concept first used by computer software developers in the 1950s [9]. Here, designs and manufacturing procedures are shared openly, typically on an online platform, from which any qualified operator can freely make, modify, or distribute a product tailored to a specific population. A common example is shared 3D printing instructions, though open-source work extends far beyond this. Open-source approach to medical technology dissemination covers the complete range of tasks required to build and distribute, including making the following freely available: the underlying design files, schematics, and/or assembly instructions. The goal is to promote transparency, adaptability, and local empowerment, rather than dependence on closed proprietary systems. In this way, such devices can be constructed or created in the communities or hospitals that live and work in LRS, thereby bypassing the financial and resource strains that foreign involvement often entail. For example, repairs and replacements of open-source devices can more often be done locally and without specialized or expensive assistance, as opposed to more expensive equipment imported overseas. Abiding by the obligations of foreign stakeholders may no longer be needed given that the open-source procedures, by definition, allow for local production and modification of such devices to fit the needs of local regulations. The open-source approach has created a pathway by which technologies have been tailored to ever-growing needs in LRS. Medical technologies thus far developed via open-source include non-invasive respiratory support targeting LRS in Nigeria, Cambodia, Mozambique, and Bangladesh [10–14]. In the context of the COVID-19 pandemic, emergency mechanical ventilators [15,16] and hand hygiene and isolation gown sanitizer projects [17–20].

Taken together, open-source pathways may lead to improved cost of production, decentralization of supplies and expertise, and customizability in a way that facilitates pediatric technology innovation for austere settings and LRS [21–24]. Given the novelty of this approach, it is critical to assess the perspectives and attitudes held by healthcare providers who frequently work in technology-heavy medical sectors (i.e., intensive care) and/or LRS in order to craft impactful interventions. Therefore, in this descriptive study we sought to survey the perspectives and opinions of medical professionals on the viability of open-source applications for pediatric global health technology.

## Materials and Methods

This exploratory study and questionnaire were approved by the Mass General Brigham Institutional Review Board (IRB) (Protocol number 2022P002692) and built in compliance with SQUIRE guidelines [25]. The entire survey was developed by the authors, then critically appraised by three pediatric critical care and emergency physicians with experience in global health and low-cost medical devices. Suggested improvements included providing an introduction to the survey and the study and adding more clarifying questions (i.e., “Please list known open-source pediatric medical devices.”). The survey was then refined, edited, and ultimately approved by the respective scientific review committees of the World Federation of Pediatric Intensive and Critical Care Societies (WFPICCS) and the Pediatric Acute Lung Injury and Sepsis Network (PALISI) (See Supplemental File “S3 File Survey Creation Timeline”) – 2 academic groups that jointly represent global pediatric critical care efforts (see Supplemental Document “Survey Creation Timeline”). WFPICCS is an international network of 52 national, international, and regional member societies that represent pediatric and neonatal critical care practitioners from Africa, Asia, Europe, Latin America, the Middle East, North America, and Oceania. PALISI is a network of research investigators involving over 90 pediatric intensive care units (PICUs) in the U.S. and Canada seeking to optimize the care of critically ill children. Critical care practitioners were selected as respondents due to their unique position of recognizing patient need, often navigating complex medical infrastructure including delivery systems, funding streams, and high-level use of medical equipment. Potential respondents received an email from WFPICCS or PALISI, which included a description of the survey and link to the survey housed on REDCap (Research Electronic Data Capture). Reminder emails were sent at 4 and 8 weeks after the initial email. The survey was active for a total of 3 months, from March 6, 2023, to June 1, 2023.

Survey questions included multiple choice, free text, Likert, and checkbox (with ‘Choose more than 1,’ options) formatting. Occupation options included “physician”, “nurse”, “advanced practice provider”, “respiratory specialist”, or “other”. Questions asking about locations (i.e., countries) and devices were free text. Questions asking about factors to consider when implementing open-source devices and barriers to implementation provided discrete answer choices but also provided an “other” option. Likert-style questions were used when asked about comfort, feasibility, and having ethical concerns regarding open-source technology implementation in LRS.

Respondents were informed on the survey that no sensitive or identifying information would be collected in the survey other than the country of practice and that voluntary completion of the online survey would constitute implied consent. The questionnaire contained 17 questions that captured data on a respondent’s experience with and knowledge about open-source pediatric medical devices and opinions on the feasibility of utilizing the open-source approach. [Survey copy available in Supplement]

Study data were collected and managed using REDCap hosted at Mass General Brigham [26,27]. REDCap is a secure, web-based software platform designed to support data capture for research studies, providing: 1) an intuitive interface for validated data capture; 2) audit trails for tracking data manipulation and export procedures; 3) automated export procedures for seamless data downloads to common statistical packages; and 4) procedures for data integration and interoperability with external sources. Statistical analysis, performed in REDCap, included descriptive analysis and calculations of proportions.

## Results

A total of 148 surveys were at least partially completed by respondents, of which 47 were incomplete, resulting in 101 (68.2%) completed surveys. Only completed surveys were used in the analysis to ensure consistency across variables. Among the 101 completed surveys, 90 (89%) were completed by physicians and 87 (86%) by providers identified as pediatric critical care specialists. Fifty-five respondents (55%) practiced primarily in the United States of America (USA); and more than 1 provider responded from Brazil, the United Kingdom, India, Kuwait, Peru, and Argentina; and single providers responded each from 27 other countries. (Table 1)

**Table 1:** Respondent characteristics among the 101 completed surveys. The “Other” category included Algeria, Bolivia, Cameroon, Canada, Chile, Colombia, Croatia, the Czech Republic, El Salvador, France, Ghana, Honduras, Japan, Lebanon, Madagascar, Malawi, Mexico, Mozambique, New Zealand, Nigeria, North Macedonia, Pakistan, Panama, South Africa, Tanzania, Netherlands, and Tunisia. Percentages reported for the total count are among all survey respondents. Percentages reported for the survey questions are among the stated profession, specialty, or home country. GH = global health.

Category	Count (%)	Any Pediatric GH Experience? (count (%))	Any Pediatric Device Experience? (count (%))	Aware of Open-Source Devices? (count (%))	Participated in Access to Devices? (count (%))
Occupation Physician Nurse Advanced Practice Practitioner	90 (89) 5 (5) 6 (6)	26 (26) 0 0	15 (17) 0 1 (17)	5 (6) 1 (20) 1 (17)	15 (17) 1 (20) 2 (33)
Medical Specialty Critical Care Neonatology Hematology-Oncology General Practice Emergency Medicine Nephrology Other	87 (86) 3 (3) 3 (3) 2 (2) 2 (2) 1 (1) 3 (3)	22 (25) 1 (33) 1 (33) 1 (50) 0 1 (100) 0	14 (16) 1 (33) 0 0 0 0 1 (33)	6 (7) 0 1 (33) 0 0 0 0	15 (17) 1 (33) 1 (33) 0 0 1 (100) 0
Country of practice USA Brazil United Kingdom India Kuwait Peru Argentina Other	55 (55) 6 (6) 3 (3) 3 (3) 2 (2) 2 (2) 2 (2) 27 (27)	18 (33) 0 2 (67) 0 0 0 0 6 (22)	8 (15) 1 (17) 2 (67) 0 0 0 0 5 (19)	4 (7) 0 0 0 0 0 0 3 (11)	8 (15) 1 (17) 0 0 0 1 (50) 0 8 (15)

USA = United States of America

**Fig 1:**
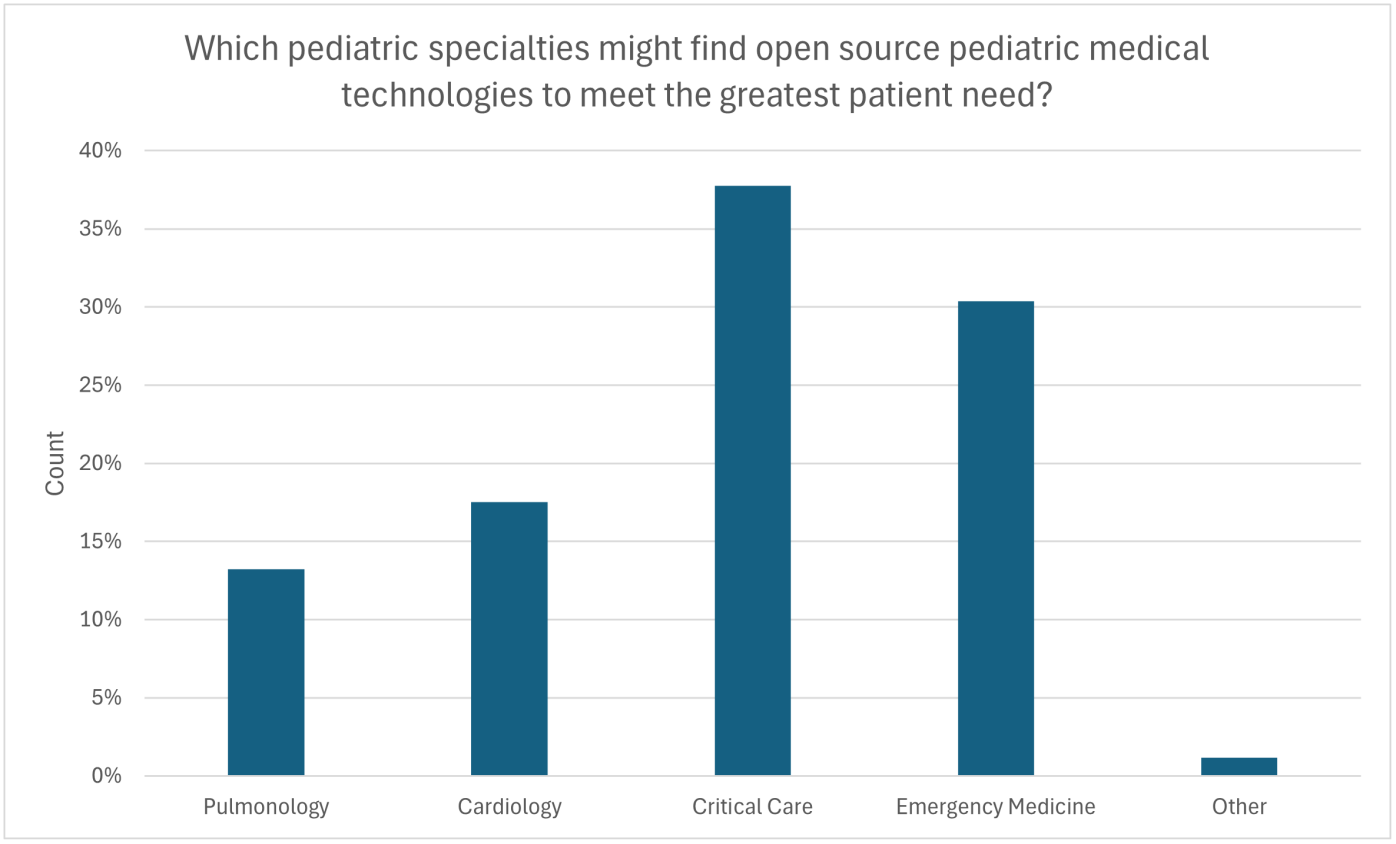
The selection counts of specialties thought to benefit the most from open-source pediatric medical device technology. The “Other” category comprised of “Oncology” once and “Nephrology” two times.

Most of the respondents felt “critical care” (n = 81, 80.2%) and “emergency medicine” (n = 66, 65.4%) would benefit the most from open-source devices (Fig 1). Of note, “Nephrology” was not provided as a defined answer option but was added by two respondents in the “Other” category as a specialty that would benefit. Sixty-nine (68.3%) of the respondents work in high-income countries (HIC), and 32 (31.7%) work in a low- or middle-income country, as defined by the World Bank [28]. Seventy-five (74.3%) reported no experience working in “pediatric global health”, but the remaining 26 (25.7%) reported working in “pediatric global health,” of whom 11 (10.9%) also had experience working with pediatric medical devices. Overall, 16 (15.8%) respondents had prior experience working with pediatric medical devices and only 7 (6.9%) reported being aware of an example of an open-source pediatric medical device. Of the 10 examples of open-source devices provided, 4 were general terms (“prosthetic limbs”, “bubble CPAP”, “PPE”, “PALS devices”), 4 were open-source devices or groups (i.e., NeoTree, Glia Project, PVP1 ventilator, Goodscope), and 2, while low-cost-oriented, were not open-source devices or groups (IMPALA continuous monitoring system, Layco Medical).

“Device durability” (n = 58, 57%) and “local institutional need and interest” (n = 57, 56.4%) were identified as the most important factors for operationalizing a device in LRS, while “government involvement” was thought to be the least important (n = 16, 15.8%). A total of 22 (21.8%) respondents had previously participated in attempts to increase accessibility to pediatric medical devices and, among this subgroup, perceived the greatest barrier to be “lack of funding” (n = 16, 72.7%) followed by “lack of local technical expertise and support” (n = 12, 54.5%). Thirteen of these 22 (59%) respondents practiced outside of the USA.

When comparing responses about implementation factors and barriers (e.g., ‘believing that device durability is a major factor’, and ‘lack of funding is a major barrier’), answers were similar between the subgroup with pediatric global health experience (n = 26) and the subgroup with device experience (n = 16). Also, of note, the subgroup with device experience marked “lack of local material supplies” as less important of a barrier and “regulatory or policy restrictions” as a greater barrier.

**Fig 2:**
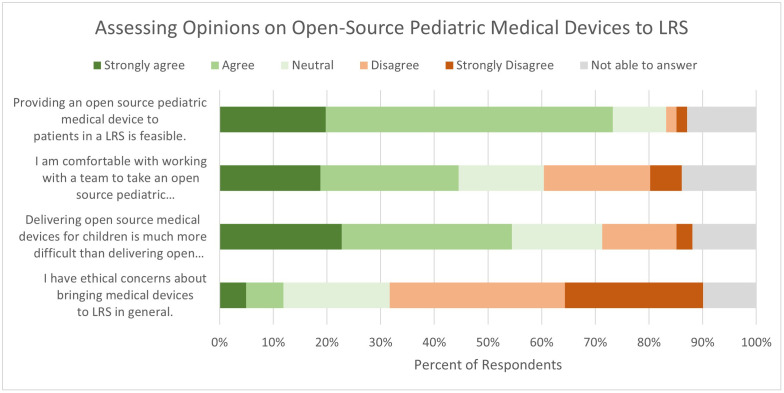
Percentage of responses to Likert questions regarding open-source pediatric medical devices. LRS = low-resource setting.

Fig 2 depicts the results from the Likert scale questions. The majority agreed or strongly agreed that providing an open-source pediatric medical device to LRS is feasible (n = 74, 73.2%); that delivering devices to children is particularly difficult (n = 55, 54.4%); and that ethical concerns were not a factor (n = 59, 58.4%). More respondents were comfortable working with a team to take a device from conception to market versus respondents who were not comfortable (45 vs 26). Of note, among the 45 who were comfortable taking a device from conception to market in an LRS, only 17 (37.7%) of them practiced in the USA. In contrast, 26 indicated they “strongly disagree” or “disagree” with being comfortable in taking a device from conception to market in an LRS, with 21 (81%) identifying as working in the USA. When asked if the respondent had ethical concerns with bringing devices to LRS, no one from the USA agreed or strongly agreed, but 12 respondents from outside the USA agreed or strongly agreed.

## Discussion

In this international survey of 101 providers from 34 countries, we sought to identify the opinions about and gaps in experience with open-source medical hardware development and distribution in LRS. Though the majority of collected responses were authored by critical care physicians without experience working with open-source devices in LRS, many respondents felt comfortable with successfully providing an open-source pediatric medical device to LRS. Among those who had experience with pediatric medical devices, most felt that lack of funding was the most significant barrier to successfully deploying technologies in LRS.

Most respondents felt that local need assessment was one of the most important factors when considering increasing access to open-source devices in LRS. These sentiments are consistent with recent global health literature that has emphasized the importance of conducting local needs assessments in the context of global health initiatives, emphasizing decolonialization, refocusing on the needs of the country, and promoting local ownership of health initiatives [29–31]. Lack of funding was identified as a significant barrier to providing open-source devices, highlighting the need for investment in cost-effective strategies or mechanisms for implementation. Examples of such efforts include the national Food and Drug Administration’s (FDA) Pediatric Device Consortia Program, although there is no precedent for analogous efforts in LRS, to our knowledge [32]. Extending a similar mechanism to LRS is needed.

Our survey results demonstrate a discrepancy between experience and comfort in approaching open-source pediatric devices for LRS. Forty-five respondents (44.5%) felt comfortable in this endeavor; however, only 11 of these 45 respondents reported having experience with devices in LRS. There may be a mismatch of the expectations and reality of attempting to implement open-source technology in LRS. For example, perhaps some practitioners with limited experience in the regulatory and development processes that take place before, during, and after a device is deployed underestimate the associated challenges. It is notable that most respondents who were comfortable taking a device from conception to market were from outside the USA, suggesting that providers in target LRS countries are more motivated to advance much needed technologies. Regardless, education and training in device development, manufacturing, and distribution should be integrated into global health didactic programs, wherein, according to our survey results, ‘device durability’ and ‘local needs assessment’ should be core concepts.

We found it interesting that most respondents with experience in “pediatric global health” were from the USA, but most respondents with experience with pediatric medical devices were from outside the USA. In this survey, we realize that “pediatric global health” may be viewed differently, based on the location of practice. Therefore, though some providers may not have experience in what they may call “pediatric global health”, they may already work in a low-resource setting. This survey element may also reflect that the type of global health work typically pursued by USA-based critical care practitioners does not involve the use of medical devices.

Our survey found that practitioners based in the USA had no ethical concerns with bringing devices to LRS. Conversely, the 15 respondents who agreed (i.e., had ethical concerns) were all from outside the USA. This discrepancy underscores what has been shown previously: that medical practitioners in high-resource countries (HIC) and LRS have differing opinions on what it means to achieve equity in global health practice [33]. For example, HIC investigators studying the implementation of new technology in a LRS may disproportionately allocate credits (leadership, authorship, funding) to the HIC investigators in the process. The perspectives and needs of the leaders in the LRS may be overshadowed by the priorities of the HIC team, which may promote the use of economic or academic pressures to control or influence the LRS despite best intentions. Such divergence may reflect deeper historical or systemic asymmetries. Many LRS practitioners may remain acutely aware of legacies of colonialism and resource extraction, which can shape their concerns about dependency, exploitation, and inequitable benefit-sharing. In contrast, practitioners in HICs may be more insulated from these narratives. There is, therefore, an opportunity for shared learning and strategizing among partners for ways to minimize any ethical tension when approaching medical device distribution to LRS [34]. Furthermore, it is important that individuals representing a local population independently support the development of the target technology in these partnerships. Of note, we did not include any queries in the survey to further delineate the interpretation of “ethical concerns”.

Our study has a few noteworthy limitations. First, the survey was limited to mostly critical care practitioners through two critical care networks. New technologies can certainly be applied by providers from other specialties or with specific research backgrounds (which was not asked) and should be explored in future investigations. While WFPICCS and PALISI are considered among the largest networks of pediatric critical care providers in the world, future investigations should be sent outside of these two organizations to better capture the full breadth of providers in LRS (membership to said societies often requires resources – internet access and funding – to participate). Future related work may also consider surveying the perspectives of professionals outside the critical care specialty, professionals who are more frequently at bedside (i.e., nurses), or those who work with other aspects of device development, such as engineers or public health workers, who may have additional insight into other aspects of device implementation. Indeed, the fact that a minority of respondents were aware of any open-source devices highlights that additional information is needed to more accurately capture information on how open-source approaches are viewed by relevant professions. Additionally, despite an attempt to capture a global audience, almost half of the respondents practiced in the USA and two-thirds of respondents who had experience in global health are based in the USA. This potentially reflects the current geographic distribution of pediatric critical care providers in the world [35]. However, determination of this in our study is limited by our low response rate. This may have been due to a lack of interest or familiarity with the topic. Indeed, no previous studies or evaluations have been published documenting the lack of open-resource devices in LRS – only that interest is growing despite multiple barriers to these efforts. Our survey reflects this gap given that most respondents did not have experience working either in pediatric global health or with open-source medical devices in LRS, which may limit how some of these results may be interpreted. For example, most respondents agreed that assessing the local need is crucial to implementing such technology, but it may not be an accurate sentiment if it reflects opinions of those with little experience. Relatedly, given the potential variability in how participants perceived the meaning of “global health” or “pediatric medical device”, future work should involve a guiding definition of both terms to ensure consistent understanding among the respondents regardless of geography or medical scope. Lastly, our survey was only provided in English.

## Conclusion

According to this exploratory study comprised of responses largely representing critical care providers in high-resource settings, most pediatric practitioners worldwide are unaware of or lack experience with open-source pediatric medical devices in LRS, with significant geographic variability. Our results reflect a disparity that magnifies the disconnect between provides in LRS and those in HIC – individuals who work in ICU settings in LRS may have little experience with appropriate technologies and, conversely, providers in HIC with greater access to technologies may not have experience working in LRS. This reveals a potential unexplored frontier in addressing inequities in health care by developing equipment and technologies with high accessibility in areas of the world with the highest burdens of treatable pediatric disease. Based on this survey data, next steps may include expanding the survey to a larger audience representing LRS, generating appropriate funding streams for development efforts, and facilitating international discussion and training on this topic given varying levels of comfort and experience with the implementation of pediatric medical devices in LRS.

## Supporting information

S1 File SQUIRE guidelines checklist. (DOCX)

S2 File Open source medical device blank survey. (PDF)

S3 File Survey creation timeline. (DOCX)

S4 File Survey raw data. (CSV)
