# Supplementary material for: Enhancing healthcare equity by using open-source pediatric medical devices in low resource settings: An exploratory international survey of pediatric clinicians
Source: PLoS One. 2025 Oct 24;20(10):e0334108. doi: 10.1371/journal.pone.0334108 (PMC12551840; doi:10.1371/journal.pone.0334108)
Supplement: S1 File — (DOCX) [file pone.0334108.s001.docx]

| **Title and Abstract** | **Completed? (Y or N/A)** | **Completed on page no.** |
| --- | --- | --- |
| Title | Y | 1 |
| Abstract | Y | 2 |
| **Introduction** |  |  |
| Problem Description | Y | 2 |
| Available Knowledge | Y | 3 |
| Rationale | Y | 3 |
| Specific Aims | Y | 3-4 |
| **Methods** |  |  |
| Context | Y | 4 |
| Interventions | Y | 4-5 |
| Study of the Interventions | N/A (survey was not expected to produce direct measured impact or outcomes) |  |
| Measures | Y | 4 |
| Analysis | Y | 5 |
| Ethical Considerations | Y | 4 |
| **Results** |  |  |
| Results | Y | 5-7 |
| **Discussion** |  |  |
| Summary | Y | 8 |
| Interpretation | Y | 8-10 |
| Limitations | Y | 10 |
| Conclusions | Y | 10-11 |
| **Other information** |  |  |
| Funding | Y | 12 |
