# Supplementary material for: Enhancing healthcare equity by using open-source pediatric medical devices in low resource settings: An exploratory international survey of pediatric clinicians
Source: PLoS One. 2025 Oct 24;20(10):e0334108. doi: 10.1371/journal.pone.0334108 (PMC12551840; doi:10.1371/journal.pone.0334108)
Supplement: S2 File — (PDF) [file pone.0334108.s002.pdf]

Open Source Pediatric Medical Devices: An International Survey of Perspectives

Globally, pediatric morbidity and mortality remain highest in low- and middle-income countries (LMICs) such as those in Sub-Saharan Africa and Southeast Asia where the gross national income is less than $4000 per capita. In order to learn more about ways to combat these trends, researchers from Massachusetts General Hospital for Children and Boston Children's Hospital are conducting a study entitled: Open Source Pediatric Medical Devices: An International Survey of Perspectives. This survey aims to collate the perspectives of relevant practitioners on the feasibility of increasing access to pediatric medical devices via open source designs in these parts of the world. We propose that open source principles - premised on collaborative design, local production, and maximal access - have the potential to accelerate pediatric global health technologies.

Specifically, "open source" refers to tools that people can modify and share because its design if publicly available. Examples of open source tools include low-cost ventilators powered by Arduino microcontrollers; WHO-style bubble CPAP; and 3D printed stethoscopes. To our knowledge, while there are multiple opinion papers and advocacy manuscripts on the topic, a global survey of experts has not been done, especially in the COVID or post-COVID era.

We are contacting you because you have been identified as a medical provider caring for children in an intensive care unit (ICU) and member of a society that subscribes to the World Federation of Pediatric Intensive and Critical Care Societies (WFPICCS) or the Pediatric Acute Lung Injury and Sepsis Investigators (PALISI). We seek to elicit your general viewpoints on the feasibility of open source pediatric medical devices in low-resource settings.

Participation in this study is entirely voluntary and confidential. Participants have the right not to participate and/or not to answer any of the questions. We greatly appreciate your willingness to complete this survey. Willingly filling out this survey acts as consent for us to analyze the data. We estimate that completion of the survey will take approximately 5 to 10 minutes. No specific personal identifying data is collected in this survey other than country of practice. The web server employed is SSL encrypted to prevent tampering with collected data. IP addresses will not be collected and none of the data collected will be used to identify individuals or organizations.

Should you have any questions regarding this survey please feel free to contact the Principal Investigator at rcarroll4@mgh.harvard.edu. Thank you for your time.

What is your occupation?

- ◯ Physician
- ◯ Nurse
- ◯ Advanced Practice Provider
- ◯ Respiratory specialist
- ◯ Other

If other profession, please state here: ________________________________

Specialty

- ◯ General practitioner
- ◯ Critical care
- ◯ Cardiology
- ◯ Neurology
- ◯ Emergency Medicine
- ◯ Endocrinology
- ◯ Gastroenterology
- ◯ Hematology-Oncology
- ◯ Infectious Disease
- ◯ Neonatology
- ◯ Nephrology
- ◯ Pulmonary
- ◯ Other

If other specialty, please state here: ________________________________

Country in which you primarily practice ________________________________

In your opinion which pediatric specialties or disciplines might find open source pediatric medical technologies the most beneficial (i.e. meeting the greatest patient need)?	☐ Pulmonology<br>☐ Cardiology<br>☐ Critical Care<br>☐ Emergency Medicine<br>☐ Other
If other, please state here:	__________________________________
Have you had experience working in countries with or within the context of PEDIATRIC GLOBAL HEALTH?	◯ Yes<br>◯ No
Please a) list the countries you have had experience working with or within the context of PEDIATRIC GLOBAL HEALTH and b) for how many total years in each country.	____
Have you had experience working in countries with or within the context of PEDIATRIC DEVICES?	◯ Yes<br>◯ No
Please a) list the countries you have had experience working with or in within the context of PEDIATRIC DEVICES and b) for how many total years in each country.	____
Are you personally aware of any open source pediatric medical devices?	◯ Yes<br>◯ No

Please give us examples of open source pediatric medical devices of which you are personally aware.

Name of Device How did you learn about it? Institution/Sponsor, if known Reference, if known (website, manuscript, etc.)

______ ______ ______ ______
______ ______ ______ ______
______ ______ ______ ______
______ ______ ______ ______
______ ______ ______ ______
______ ______ ______ ______

This is a list of factors that may be important to consider while operationalizing open source devices in LMIC's. Which, in your opinion, are the THREE most important? If you are unable to answer, please check "Not able to answer".	☐ Local institutional need and interest (e.g. local physicians or administrators)<br>☐ Device durability (e.g. sturdy working parts, materials resistant to changes in humidity, temperature)<br>☐ Device production quality control and patient safety<br>☐ Existing international working partnerships (e.g. university to university partnership, non-profit networks)<br>☐ Manufacturing process (e.g. local technicians, local production capacity, local supplies)<br>☐ Manufacturing monetary costs (e.g. supplies, construction)<br>☐ Government involvement<br>☐ Other<br>☐ Not able to answer

If other, please state here

____

Have you participated in attempts to increase accessibility to pediatric medical devices?

- ○ Yes
- ○ No

Which barriers have you personally encountered when trying to increase accessibility to pediatric medical devices in LMICs? Please mark THREE.

- ☐ Lack of funding
- ☐ Lack of local material supplies
- ☐ Lack of local technical expertise or support
- ☐ Poor device quality
- ☐ Lack of established inter-institutional or inter-organization relationship
- ☐ Regulatory or policy restrictions
- ☐ Local needs are unclear or unable to be ascertained
- ☐ Other

If other, please state here

____

	Strongly disagree	Disagree	Neutral	Agree	Strongly agree	Unable to answer
Providing an open source pediatric medical device to patients in a LMIC is feasible.	○	○	○	○	○	○
I am comfortable with working with a team to take an open source pediatric medical device from conception to market for a LMIC.	○	○	○	○	○	○
Delivering open source medical devices for children is much more difficult than delivering open source devices for adults.	○	○	○	○	○	○
I have ethical concerns about bringing medical devices to LMICs in general.	○	○	○	○	○	○
