# Supplementary material for: Enhancing healthcare equity by using open-source pediatric medical devices in low resource settings: An exploratory international survey of pediatric clinicians
Source: PLoS One. 2025 Oct 24;20(10):e0334108. doi: 10.1371/journal.pone.0334108 (PMC12551840; doi:10.1371/journal.pone.0334108)
Supplement: S3 File — (DOCX) [file pone.0334108.s003.docx]

**Open-Source Pediatric Medical Device Survey – Creation and Validation Timeline**

July 2022 - The survey was initially written by the study authors, including questions about barriers to implementation of devices, importance of decisional factors, and examples of known open-source devices.

July 2022 - August 2022 - The survey was sent to a small group of pediatric ICU and pediatric emergency medicine physician colleagues for pilot testing.

August 2022 - October 2022 - The survey was sent to the World Federation of Pediatric Intensive and Critical Care Societies (WFPICCS) for feedback and afterwards, distribution. The WFPICCS scientific review board responded with several questions to which we responded.

- Feedback included the following:
  - Significant rewording of the introduction paragraph to the survey, including a definition of “open-source”, a mention of IRB approval, and information about implied consent
  - Including general pediatricians and other professions who may practice critical care in LRS

September 2022 - December 2022 - The survey was sent to the Pediatric Acute Lung Injury and Sepsis Investigators (PALISI) for review and feedback. Two scientific reviewers responded to us with requested edits with a total of 18 comments over two review interactions.

- Feedback included the following:
  - Providing preset options for questions instead of allowing free text
  - Adding a question about the participant’s specialty
  - Allowing non-answers (i.e. not knowing any open source devices)
  - Adding how the respondent learned about a device
  - Adding a question about global health experience, separate from device experience
  - Asking for the number of years involved with a certain country
  - Adding specifying language to the decisional factors of device implementation work
  - Added more options for possible decisional factors of device implementation work
  - Reducing wording redundancy in the Likert scale questions
  - Adding a question about their specific occupation (physician, nurse, respiratory, etc.)
